# Hydrogen-assisted combustion of GQD–DEE-enhanced WCO biodiesel in a dual-fuel CI engine

**DOI:** 10.1186/s11671-026-04843-9

**Published:** 2026-08-03

**Authors:** Vinod Kumar Mallipudi, S. J. Margarette, Golamari Siva Reddy, Janaki Durga Venkatesh, Debabrata Barik, Prabhu Paramasivam, D. Shanmugapriya, Abinet Gosaye Ayanie

**Affiliations:** 1 Department of Mechanical Engineering, Sasi Institute of Technology and Engineering, Tadepalligudem, 534101 India; 2 Department of Physics (BS&H), Malla Reddy Engineering College for Women (Autonomous), Secunderabad, Telangana 500100 India; 3 Department of Bio Technology, Koneru Lakshmaiah Education Foundation, Green Fields, Vaddeswaram, Guntur, 522502 India; 4 Department of Mechanical Engineering, Aditya University, Surampalem, 533437 India; 5 Department of Mechanical Engineering, Karpagam Academy of Higher Education, Coimbatore, 641021 India; 6 Centre for Energy and Environment, Karpagam Academy of Higher Education, Coimbatore, 641021 India; 7 Centre for Research Impact & Outcome, Chitkara University Institute of Engineering and Technology, Chitkara University, Rajpura, Punjab 140401 India; 8 Vicerrectoría de Investigación y Postgrado, Universidad de La Serena, 1700000 Código, Chile; 9 Department of Mechanical Engineering, Adama Science and Technology University, 1888 Adama, Ethiopia

**Keywords:** Clean energy, Emission reduction, Waste utilization, Nano-graphene quantum dots, Hydrogen, Ternary fuel

## Abstract

The relatively low performance and elevated nitrogen oxide (NOx) emissions associated with biodiesel-fueled compression ignition (CI) engines continue to limit their widespread application. To address these challenges, the present study investigates the combined influence of diethyl ether (DEE), graphene quantum dots (GQDs), and hydrogen enrichment on the performance, combustion, and emission characteristics of a dual-fuel CI engine. Waste cooking oil (WCO)-derived biodiesel was blended with diesel at a ratio of 20:80 (B20) and supplemented with 10 vol.% DEE to formulate a ternary fuel (TF) blend. GQDs were dispersed into the TF blend at a concentration of 50 mg/L using an ultrasonicator (Hielscher UP400S, 160 W, 40 kHz) to ensure stable and uniform dispersion. The physicochemical characteristics of the synthesized GQDs were analyzed using Fourier Transform Infrared Spectroscopy (FTIR), Field Emission Scanning Electron Microscopy (FESEM), and High-Resolution Transmission Electron Microscopy (HRTEM). Hydrogen (H2) was introduced through the intake manifold at flow rates of 5 and 10 LPM, serving as a supplementary fuel. The results demonstrated that the TF + GQD50 + 10H_2_ combination yielded the best overall performance. Compared with the baseline fuel, brake thermal efficiency increased by 13.7%, while brake specific fuel consumption decreased by 20.46%. Furthermore, Cylinder Pressure (CP) and Heat Release Rate (HRR) improved by 29.47% and 4.49%, respectively. Significant reductions in Carbon monoxide (CO), Hydrocarbons (HC), NOx, and smoke emissions of 17.52%, 11.01%, 3.90%, and 2.22% were also observed at higher brake power. These findings indicate that the combined use of DEE, GQDs, and hydrogen can effectively enhance biodiesel combustion and emission characteristics, although long-term durability and practical implementation require further investigation.

## Introduction

The global energy system faces mounting pressures from rapid economic expansion, accelerating industrialization, population growth, urbanization, and rising living standards—particularly in emerging and developing economies [1]. According to the International Energy Agency's (IEA) World Energy Outlook 2025, global primary energy demand grew by 2.2% in 2024, exceeding the average annual growth rate of 1.3% from 2013 to 2023 [2]. Projections indicate continued increases through the coming decades, though at varying rates depending on policy pathways. In the Stated Policies Scenario (STEPS), total energy demand rises by approximately 8% (around 55 exajoules) by 2035 relative to recent levels [3]. As resource depletion advances and environmental pressures intensify, the swift development, large-scale deployment, and seamless integration of renewable energy alternatives alongside efficiency improvements and diversified supply chains become essential [4]. This transition is critical to establishing secure, affordable, low-carbon energy systems that can equitably support global development amid escalating demands while averting irreversible environmental consequences.

Out of many alternative energy resources, biodiesel has been explored as a strong alternative and sustainable energy source. Biodiesel is more popular due to its biodegradability, environmental friendliness, and the fact that its feedstock is obtained from various resources such as vegetable oils, waste oils, animal fats, microalgae, and microorganisms [5]. The huge availability of these feedstocks facilitates large-scale production, which already exists in different countries, including the United States, Europe, and Brazil, etc. [6]. Biodiesel is prepared mostly from edible or non-edible plant-based seed oils, which contain triglycerides and Free Fatty Acids (FFA) in different ratios. These are converted into methyl esters through a well-known process called transesterification for the best yield [7, 8]. Biodiesel is derived as a mono-alkyl ester prepared from vegetable/non-edible oils via the transesterification technique with a catalyst and in the presence of alcohol. The prepared biodiesel cannot be used directly due to various problems, such as low energy content, filter clogging, poor cold flow properties, blocked fuel injectors, and higher NOx emissions. This is the reason the B20 blend is more popular, which contains 20% of biodiesel mixed with 80% of neat diesel, which is a universally accepted blend [9, 10]. The biodiesel blend in diesel fuel improves the lubricity, increases the cetane number, and reduces harmful emissions, which encourages researchers to study biodiesel-diesel blends [11, 12]. Though the B20 sample offers good benefits, a few disadvantages result from the use of different additives to overcome them, which include lower calorific value and higher NOx emissions [13].

Many scholars investigated fuel reformulation strategies, which include blending the B20 sample with alcohols, cetane improvers, antioxidant compounds, and NPs. Among these methods, nanotechnology has emerged across a wide range of fields, including industrial, medical, engineering, agricultural, biotechnological, and automotive fields [14]. The nanoparticles are found to be soluble in base fluids and improve the thermal and physical properties as compared to microparticles. The dispersion of NPs in the B20 sample improves combustion performance and lowers emission parameters due to the large surface area of NPs, superior thermal and heat diffusivity capabilities, and catalytic activity of NPs [15]. Further, the addition of NPs in the B20 sample enhances the physico-chemical properties, which accelerate the combustion phenomenon, improve performance, and reduce emissions [16, 17].

Extensive research has demonstrated that doping biodiesel or biodiesel-diesel blends with various metal and metal-oxide nanoparticles, including copper, Aluminium Oxide (Al_2_O_3_), Titanium Dioxide (TiO_2_), Iron Oxide, Cerium Oxide (CeO_2_), Silver, Boron compounds, and magnesium sulphate, significantly enhances key engine performance metrics in compression-ignition systems [18, 19]. These NPs elevate the spray jet momentum, thereby intensifying secondary atomization and promoting micro-explosion phenomena, which facilitate finer droplet breakup, superior air–fuel premixing, and accelerated evaporation [20]. Functioning as combustion catalysts, the nanoparticles leverage their high surface-to-volume ratio and inherent catalytic activity to expedite oxidation reactions, shorten ignition delay, increase HRR and CP, and achieve more complete combustion [21]. Consequently, this yields higher BTE, reductions in BSFC, and substantial decreases in exhaust pollutants such as HC, CO, and smoke opacity. However, the elevated combustion temperatures associated with enhanced reactivity often result in increased NOx emissions. In the current study, GQD NPs are chosen, which are characterized by their zero-dimensional quantum confinement, low toxicity, and excellent biocompatibility, offering several advantages over conventional carbon-based nanomaterials. These include tunable photoluminescence suitable for diagnostic and sensing applications, superior dispersibility, minimal agglomeration due to their nanoscale dimensions, reduced entanglement compared with one-dimensional carbon nanotubes (CNTs), enhanced stability relative to two-dimensional graphene nanoplatelets (GNPs), and lower cytotoxicity than graphene oxide (GO), making them attractive for a wide range of advanced applications [20, 21]. Studies have reported that nanoparticle concentrations in the range of 25–100 mg/L effectively enhance combustion characteristics without causing agglomeration, sedimentation, fuel filter blockage, or excessive viscosity. Therefore, 50 mg/L of GQD NPs was chosen as an optimum concentration to achieve improved catalytic activity, thermal conductivity, and fuel oxidation while maintaining fuel stability.

To counteract this trade-off, several investigations have incorporated oxygenated alcohol additives (e.g., Ethanol, n-Butanol, Pentanol, Dimethyl Carbonate (DMC), and DEE) into nanoparticle-blended B20 formulations, which effectively suppress peak in-cylinder temperatures through their high latent heat of vaporization and promote leaner, lower-temperature combustion regimes, thereby mitigating NOx formation while preserving or further enhancing overall performance and emission benefits [22, 23]. Literature indicates that DEE concentrations around 10% offer significant improvements in performance and emissions without causing adverse effects such as excessive reduction in fuel viscosity, lower calorific value, or ignition instability. Hence, 10 vol.% DEE was selected as an optimal proportion for the present investigation. For instance, Sakthivadivel et al. [24] explored a 1-cylinder, four-stroke, vertical engine, air-cooled, Direct Injection (DI), diesel engine employing need oil methyl ester-diesel-ethanol-DEE blends, doped with Al_2_O_3_ NPs. Very limited studies are identified using DEE enriched with ethanol in neem biodiesel along with Al_2_O_3_ NPs. The unique features of DEE result in better results in decreasing cylinder temperature and NOx emission over ethanol due to improved miscibility, superior cetane number, better cold-start ignition, improved volatility for efficient atomization, and NOx suppression via cooling. The NPs act as a catalyst, shortening ID, boosting reaction rates, and improving BTE while curbing emissions. The results show that the BTE is increased by 7.2%, and BSFC is reduced by 6.7% with the inclusion of 25 ppm Al_2_O_3_, 10% of DEE, and 20% of ethanol in Neem oil methyl ester and diesel blends. A significant reduction in HC, CO, and smoke is observed. Nevertheless, the NOx emissions are mitigated by 17.5% as compared to Neem biodiesel. In another article, conducted by Fayyaz et al. [25] studied the inclusion of manganese NPs (at a dosage of 250 mg/l, 375 mg/l, and 500 mg/l), DEE (10% Vol.) blended in diesel fuel, and tested on a 3-cylinder, four-stroke, water-cooled, DI, diesel engine. No research was carried out using the combination of manganese NPs and DEE in diesel blends to mitigate CO_2_ emissions. The DEE enhances cetane number, ID, and miscibility in diesel. While NPs catalyze combustion, boost BTE, reduce ID, and improve atomization for superior performance-emission balance. The outcomes disclosed improved BTE to 24.19–28.17%, and CO_2_ was increased by 11.57–30.52% at the optimal NPs concentration of 10% DEE + 250–500 mg Mn at 1300 rpm. Moreover, the BTE is improved to 33.06%, and CO_2_ is increased by 6.07% for 10% DEE + 375 mg Mn at 1300 rpm. In a study, EL-Seesy [26] investigated a 1-cylinder, 4-stroke, DI diesel engine to evaluate performance, combustion, and emission parameters using jojoba biodiesel-DEE (10% Vol.)- surface-modified Multi-Wall Carbon Nanotubes (MWCNTs) at a dosage of 75 ppm. The study finds DEE to be a cetane modifier due to its higher value, ignition stability, easy evaporation, and atomization, and an NOx mitigator as compared to other types of alcohols. Further, stated that the carbon-based nanoparticles MWCNTs offer superior surface area/dispersion for catalytic efficiency over metal oxides, enabling deeper emission cuts and BTE improvements without agglomeration. The outcomes of this study reveal that the addition of NPs in the Jojoba biodiesel + Diesel + DEE mix resulted in reduced CO, HC, and soot emissions by 37, 16, and 25% as compared to diesel at maximum load conditions. Whereas, CP and HRR are superior to diesel fuel. Nevertheless, the BTE is raised by 8%, and BSFC is lowered by 5% under the same circumstances. In another article, Tamrat et al. [27] experimented with the dispersion of CeO_2_ at a dosage of 75 mg/l and DEE at a Vol. Of 2% blended in castor biodiesel and run on a 1-cylinder, 4-stroke, DI, CI engine. The synergistic effect of CeO_2_ and DEE in caster biodiesel blends was not carried out in previous investigations. The addition of CeO_2_ improves catalytic oxidation, shortens delay, excels over Al_2_O_3_/Zinc oxide (ZnO) NPs via redox cycling for 20–30% deeper HC/CO/soot cuts and 10–15% BTE improvements, minimizing agglomeration. Moreover, the DEE addition surpasses ethanol with a high cetane value for stable ignition, better volatility, better atomization, greater miscibility, shorter ID, and NOx cooling without separation. The results disclosed that the inclusion of CeO_2_ and DEE in B10 and B15 showed improvement in Torque, Brake Power (BP), and BTE by 13.8, 8.6, and 14%. While HC and soot are lowered by 28% and 14%, respectively. While CO is marginally decreased, NOx is unchanged. In another study, Bikkavolu et al. [23] experimented with the addition of n-Butanol, DEE alcohols, and Graphene Oxide (GO) NPs in Nerium oleander oil-based B20 to evaluate the performance, combustion, and emission characteristics on a 1-cylinder, 4-stroke, DI, diesel engine. The effect of GO (at a concentration of 50 ppm), n-Butanol/DEE (10% Vol.) in Nerium oleander biodiesel blends was not identified. The inclusion of DEE improves volatility and cetane index over n-Butanol alcohol, while GO NPs act as a catalyst for combustion, prevent agglomeration with QPAN80 surfactant/stabilizer, reduce the ID, enhance BTE, and reduce exhaust emissions. The results showed that the blend B20GO50 + DEE10% improves the BTE, CP, and HRR by 4.91, 8.1, and 3.8% at higher BP. Moreover, the BSFC, ID, and CD are alleviated by 15.15, 20.5, and 9.3% compared to the B20 sample. Nevertheless, the emission characteristics, such as CO, HC, and NOx, are lowered by 17.4, 28, and 7% for the same mix as the B20 sample at higher BP.

Hydrogen H_2_ has emerged as a compelling alternative fuel for CI engines, driven by its unique physicochemical properties and potential to address decarbonization challenges in heavy-duty transportation. Unlike carbon-based fuels, hydrogen contains no carbon atoms, rendering it non-toxic and inherently free of CO_2_, CO, and particulate matter emissions during combustion. Its exceptionally high energy content by mass (approximately 120 MJ/kg, roughly three times that of diesel), combined with rapid flame propagation speed, wide flammability limits, and high diffusivity, enables cleaner, more efficient burning with potentially superior combustion characteristics [28]. In dual-fuel CI configurations where hydrogen is typically inducted into the intake manifold and diesel serves as the pilot ignition source, these attributes often translate to improved brake thermal efficiency, enhanced power output, and reduced specific fuel consumption at moderate to high loads, owing to faster flame velocity and more uniform mixture formation [29]. Recent investigations have demonstrated that hydrogen enrichment can significantly mitigate unburned hydrocarbons and smoke opacity while preserving or elevating overall engine performance [20, 29, 30].

However, a primary drawback remains the substantial increase in NOx emissions, primarily attributable to elevated in-cylinder peak temperatures that favor thermal NOx formation via the Zeldovich mechanism. This trade-off has spurred extensive research into mitigation strategies and synergistic fuel formulations. Notable approaches include:

- Blending diesel with biodiesel (e.g., soybean, mahua, or waste cooking oil-derived) to leverage biodiesel's oxygen content for partial NOx control while incorporating hydrogen enrichment [31].
- Ternary blends of biodiesel–diesel–alcohol (e.g., ethanol, butanol, DMC, DEE) supplemented with hydrogen to further modulate combustion phasing and reduce NOx emissions [32].
- Addition of metal oxide/carbon-based NPs (such as Al_2_O_3_, TiO_2_, CeO_2_, Carbon Nanotubes (CNTs), Graphene Nanoplatelets (GNPs), or Graphene Oxide (GO) to biodiesel–diesel blends, often combined with hydrogen induction, which enhances catalytic effects, improves atomization, and yields reductions in CO, HC, and smoke alongside performance gains [21, 30].

For instance, in research carried out by Polat et al. [33], the inclusion of CeO_2_ in sunflower oil biodiesel blends as a primary fuel, and H_2_ is supplied at a rate of 5 and 10 lpm as a supplementary fuel. The addition of nanoparticles improves the oxygen storage, reaction rate, fuel economy, and performance while cutting the emissions. Further, H_2_ is chosen for its greater diffusivity and quick burning to boost the nano fuel blend. The synergistic effects of CeO_2_ in the B20 sample and H_2_ addition favored combustion, improved overall performance, and reduced emission characteristics. The outcomes revealed that BTE is increased by 1.39–7.01% and BSFC is lowered by 1.77–7.19%. While CO, HC, and NOx are lowered by 46.51–63.57%, 12.09–28.57%, and 6.34–20.71%, for 10 lpm infused H_2_ with B20 + CeO_2_ blend. In another investigation, Selvanathan and Vijayaragavan [34] studied the addition of an underexplored aromatic alcohol (benzyl) and DEE in Calophyllum inophyllum biodiesel blends as a primary fuel and H2 as a supplementary fuel along with air on a 1-cylinder, 4-stroke, dual-fuel diesel engine. The combination of benzyl and DEE in the B20 sample and H_2_ infusion as a secondary fuel has not been explored to fulfill the research gap to improve performance parameters and mitigate emissions. The study reveals that the BTE increased by 6.54% and BSFC decreased by 28%. while emissions of CO, HC, and smoke are lowered, and NO is slightly increased. In another study, Bharti and Debbarma [35] revealed that the addition of biodegradable/waste-derived Silica Dioxide (SiO_2_) NPs (at dosages of 50, 75, 100, 125, and 150 mg/l) in karanja biodiesel blends (B20) with H_2_ infusion (at 10 lpm) was tested on a dual-fuel CI engine. The novelty of the article lies in the synergistic effect of biodegradable SiO_2_ NPs in the B20 sample and H_2_ addition with air at the intake manifold to enhance the overall engine performance and mitigate emissions. The H_2_ addition is chosen for renewability, eco-friendliness, and combustion enhancement to reduce PM while improving BTE. The SiO_2_ NPs are chosen from waste incense ash for biocompatibility, low toxicity, and catalytic effects, improving fuel stability/efficiency in biodiesel. The findings showed that the BTE is increased by 15.58%, and emissions such as CO and HC are decreased by 41.18 and 31%. While NOx emissions are marginally decreased. In another study, Zhang et al. [36] experimented with the inclusion of TiO_2_ at a quantity of 100 mg/l in WCO-based B20, used as a pilot fuel, and H_2_ as a secondary fuel at 5 lpm under a variable speed of 1800–2800 rpm and tested on a dual-fuel diesel engine. The NPs improved heat transfer and enhanced atomization and catalytic oxidation, which aid in improving combustion. H_2_ possesses high energy density and lowers emissions in dual-fuel mode. The outcomes of the study reveal that the BTE is raised by 5.2%, BSFC, CO, HC, and CO are mitigated by 8.5, 25, 30, and 15%. While NOx is increased by 8%.

### Novelty of the current study

Contemporary diesel engine research increasingly explores ternary and hybrid fuel strategies to mitigate reliance on fossil diesel while addressing performance limitations and exhaust emissions from biodiesel blends. A limited prior study has incorporated alcohols (e.g., ethanol, methanol, and DMC) or NPs such as TiO_2_, SiO_2_, CNTs, GNPs, or GO into biodiesel-diesel blends (typically B20) as the primary fuel, with H_2_ inducted along with air as a supplementary fuel in dual-fuel mode. These approaches leverage alcohol's oxygen content for improved combustion and NPs' catalytic effects for enhanced atomization and heat transfer, while H_2_ enriches the charge, promoting faster flame propagation and reduced soot/CO emissions. The current study innovates by selecting Graphene Quantum Dots (GQDs) as the nanoparticle additive and DEE as the alcohol component, blended into waste cooking oil methyl ester (WCOME)-based B20 fuel. GQDs, exhibiting zero-dimensional quantum confinement, low toxicity, and biocompatibility, provide superior advantages over conventional carbon-based nanomaterials: tuneable photoluminescence for potential diagnostic applications, exceptional biocompatibility, reduced entanglement (unlike 1-D CNTs), better dispersibility (surpassing 2-D GNPs), and lower cytotoxicity risks compared to oxidized GO. These attributes enable more uniform dispersion, improved fuel stability, and enhanced micro-explosion phenomena during combustion. On the other hand, DEE is an ether with a remarkably high cetane number (typically 125–160, far exceeding diesel's 45–55), and it offers exceptional ignition quality, high volatility, rapid evaporation, and superior atomization characteristics. Its low autoignition temperature and oxygen content further promote complete combustion, contrasting with lower-cetane alcohols like ethanol. The novel ternary fuel (TF) blend doped with GQD NPs serves as the primary injected fuel in a dual-fuel CI engine configuration, and H_2_ gas is simultaneously inducted at a flow rate of 5 and 10 lpm at the intake manifold as the secondary fuel with air. This combination has not been explored in previous investigations. This synergistic combination aims to optimize energy release, improve thermal efficiency, and minimize regulated emissions (e.g., NOx, CO, HC, particulate matter) through enhanced premixed combustion, reduced ignition delay, and cleaner oxidation pathways.

## Preparation of biodiesel

The used WCO was collected from the local market in Coimbatore, India. The used oil is contaminated and has altered its physical and chemical properties due to long-term heating, which makes it toxic to use further. Disposing of such oils may cause water pollution, which results from using this waste feedstock as a raw material for preparing biodiesel rather than disposing of it. The polar components, such as Free Fatty Acids in the WCO, are more imperative to turn WCO into biodiesel through the transesterification process [37]. Initially, the WCO is taken into a 3-neck round-bottom flask, stirred at a speed of 200 rpm, and the heater is set at 45℃. On the other side, a 1.5% wt. of Potassium hydroxide (KOH) and methanol blend was prepared and slowly poured into the WCO. The molar ratio (WCO to methanol) used in this process is 1:6. This mixture is allowed to react for 2 h under the same circumstances. After this process, the solution is allowed to cool and settle in a separating funnel. After separation, the bottom glycerol was drained, and the top methyl ester was washed with warm water and heated to avoid moisture. The prepared WCOME is found to be 97.6% based on Eq. 1 given below.

The required solvents, such as methanol, KOH pellets (85%), n-hexane (99%), and DEE, are obtained from Merck Laboratories, Mumbai, India. Moreover, Platonic Nanotech Private Limited, Kachwa Chowk, Godda District, Jharkhand, India, provided the Graphene Quantum Dots NPs as given in Table 1.

1 $$ {\mathrm{Biodiesel}}\;{\mathrm{yield}}\;{\text{(wt \% ) = }}\left[ {\frac{{{\mathrm{mass}}\;{\mathrm{of}}\;{\mathrm{biodiesel}}\;{\mathrm{(g)}}}}{{{\mathrm{mass}}\;{\mathrm{of}}\;{\mathrm{oil}}\;{\mathrm{(g)}}}}} \right] \times 100 $$

**Table 1:** The properties of the equipment adopted in GQD analysis

Analysis	Characteristics
FTIR	Perkin–Elmer, in the USA, the range of 4000–500 cm^−1^
XRD	Specs EA 10 Plus energy, radiation source of Al Ka
FESEM	JEOL (7610F Plus) electron microscope

### Formulation of fuel blends

The first sample is D100, which is neat diesel, and the second sample is B20. The third sample is named TF, which contains B20 and 10% Vol. of DEE. The fourth sample is prepared by blending surface-modified GQD at a concentration of 50 mg/l with the TF sample, which is named TF + GQD50. The GQD NPs and the most popular surfactant QPAN80 were mixed at a ratio of 1:4 with the aid of n-Hexane solvent and by employing an ultrasonicator (Hielscher ultrasonic UP400S; 160 W and 40 kHz) for 30 min, and dried afterward. The dried and surface-modified NPs are then measured at a quantity of 50 mg/l and mixed in the TF mix. The fifth and sixth samples are TF + GQD50 + 5H_2_ and TF + GQD50 + 10H_2_, in which the TF + GQD50 sample is pilot fuel to a dual fuel, diesel engine, while the H_2_ is supplied at 5 and 10 lpm with air. The physicochemical properties of the prepared fuel samples are tested as per American Society for Testing and Materials (ASTM) standards, as given in Table 2.

**Table 2:** Physico-chemical properties of D100, B20, TF, and TF + GQD50 blends

Fuel Characteristics	Testing method	Diesel Limit		Biodiesel Limit		D100	B20	TF	TF + GQD50
		ASTM D975	Petro diesel	ASTM D6751	EN14214				
Density (kg/m^3^ at 15 °C)	ASTM D1298	850	832	880	860 to 900	838	840	841	845
Kinematic viscosity (mm^2^/s @ 40 °C)	ASTM D445	2.0 to 4.5	2.84	1.9 to 6.0	3.5 to 5.0	2.92	4.0	3.7	3.8
Calorific value (MJ/kg)	ASTM D2015	42 to 46	45.3	–	35	42.6	38	40	41
Flashpoint (°C)	ASTM D93	60 to 80	69.2	100 to 170	120 min	70	90	83	90
Fire point (°C)	ASTM D93	–	72	–	–	75	94	88	95
Cloud point (°C)	ASTM D2500	− 35 to + 15	2	− 3 to − 12	–	2.0	1	− 1	− 2
Pour point (°C)	ASTM D97	− 15 to + 5	1	− 15 to − 16	–	1.0	− 4	− 5	− 6

### Hydrogen infusion in diesel engines

H_2_ is a carbon-free, clean energy carrier and a promising renewable fuel option compared to biofuels, Compressed Natural Gas (CNG), and Liquefied Petroleum Gas (LPG) in terms of environmental benefits. H_2_ enables near-zero direct emissions (only water vapor upon combustion) in various fields such as electric power generation, industry, and the transportation sector. Its advantages include minimal noise/vibration in appropriate systems, superior combustion characteristics, and reduced pollutant emissions in engines. Hydrogen's lower calorific value is 120 MJ/kg, which far exceeds gasoline (~ 44–47 MJ/kg) and diesel (~ 42–45 MJ/kg) [32]. While its effective knock resistance supports high-efficiency operation in spark-ignition contexts. As a supplementary fuel in Spark Ignition (SI) and CI engines, hydrogen is inducted via the manifold at regulated flow rates, promoting homogeneous mixtures, faster flame propagation, improved BTE, and lower CO, HC, and particulate emissions (though NOx may rise without mitigation). Physico-chemical properties of H_2_ are given in Table 3. Hydrogen serves as a valuable supplementary fuel in dual-fuel engines, particularly nano-biodiesel fuel blends-hydrogen configurations. Thus, enabling sustainable transitions by significantly reducing CO_2_, CO, HC, and particulate emissions, while leveraging existing engine infrastructure as a bridging technology toward full decarbonization and widespread adoption of green hydrogen.

**Table 3:** Shows the phytochemical properties of H_2_

Property	Value	Units
Calorific value	120	MJ/kg
Density	0.082	kg/m^3^
Adiabatic flame temperature	2475	K
Flame velocity	1.84	m/s
Self-ignition temperature	855	K
Purity	99.9	%

## Characterization of graphene quantum dots

### FTIR analysis

**Fig. 1:**
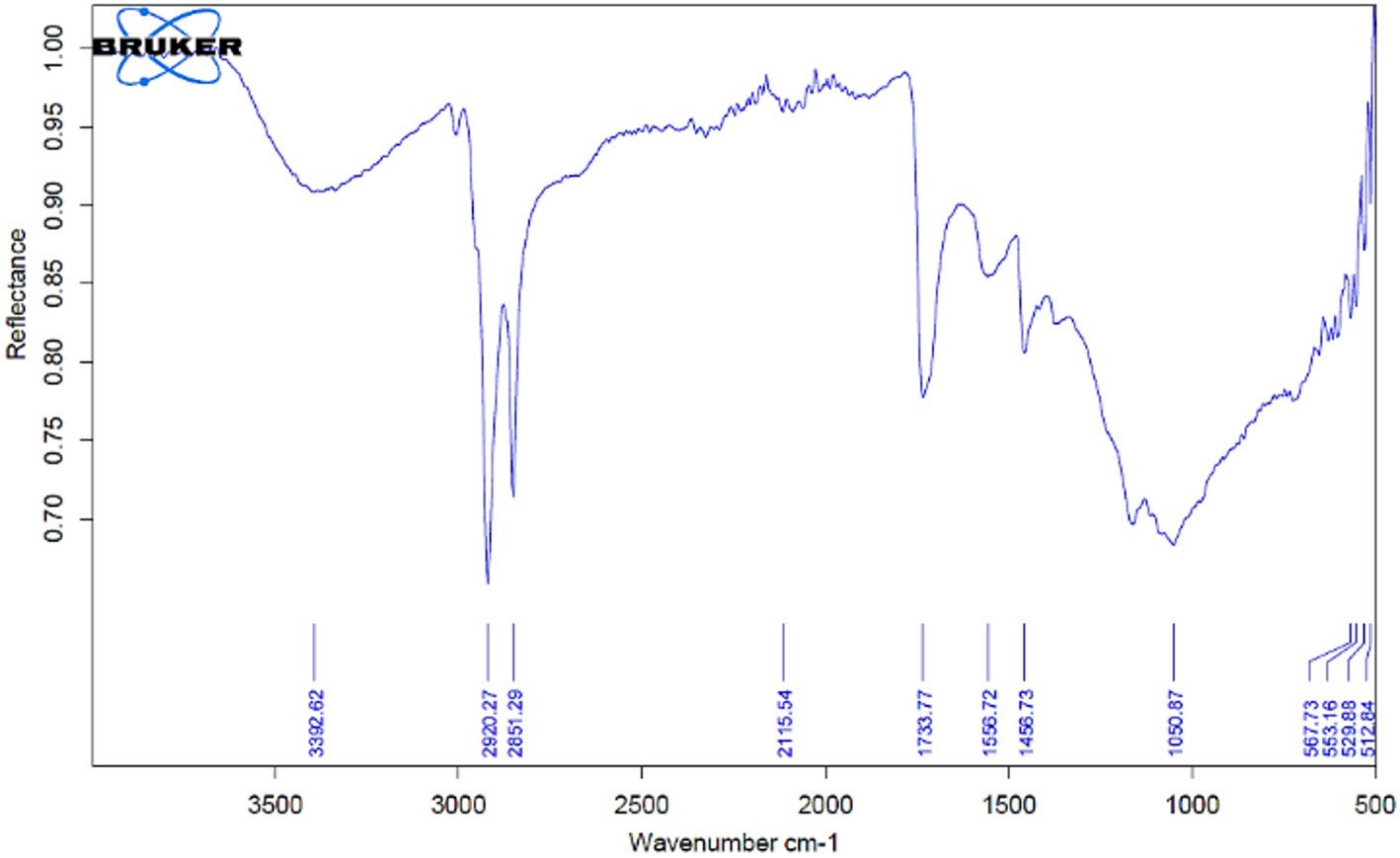
FTIR analysis of GQD-synthesized nanoparticles

The chemical structure of GQDs was characterized using FT-IR spectral analysis. The FT-IR spectrum recorded for the GQD sample appears in Fig. 1. Functional groups, including carboxyl, hydroxyl, and epoxy, are present on the GQD sheets. Figure 1 displays the FTIR spectrum of the QDs. Absorption bands located at 1733.77, 1556.72, and 1456.73 cm^−1^ correspond to C = O stretching, OH deformation, and C–O stretching vibrations of COOH groups [38, 39]. The C–O stretching vibration associated with epoxide groups appears at 1050.87 cm^−1^. Peaks observed at 1620 cm^−1^ and 3392.62 cm^−1^ are assigned to aromatic C = C stretching and O–H stretching of carboxylic groups, respectively. Additionally, CH stretching absorption is visible at 2920.27 cm^−1^ [38].

### XRD analysis and FESEM

**Fig. 2:**
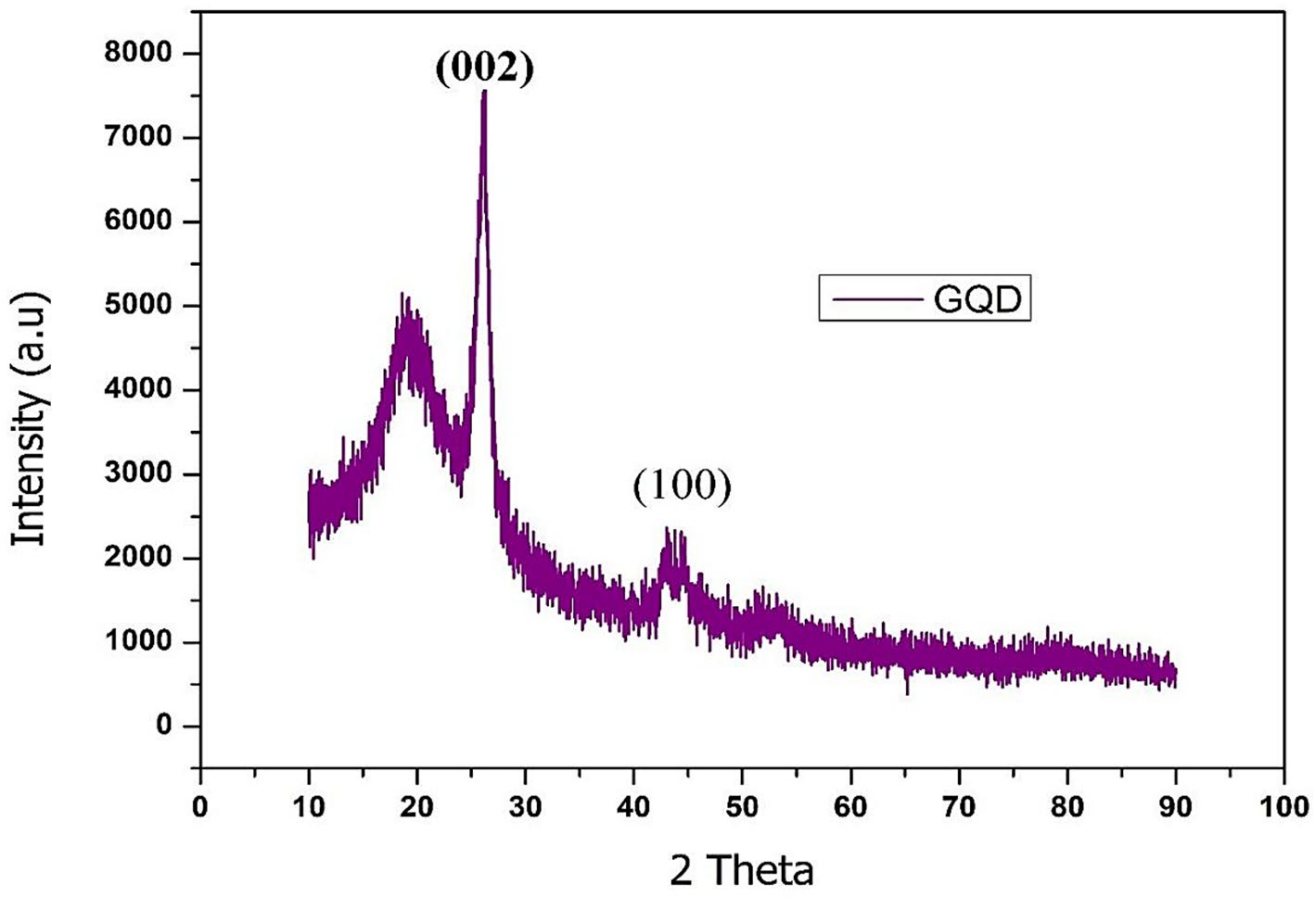
XRD analysis of GQD synthesized nanoparticles

**Fig. 3:**
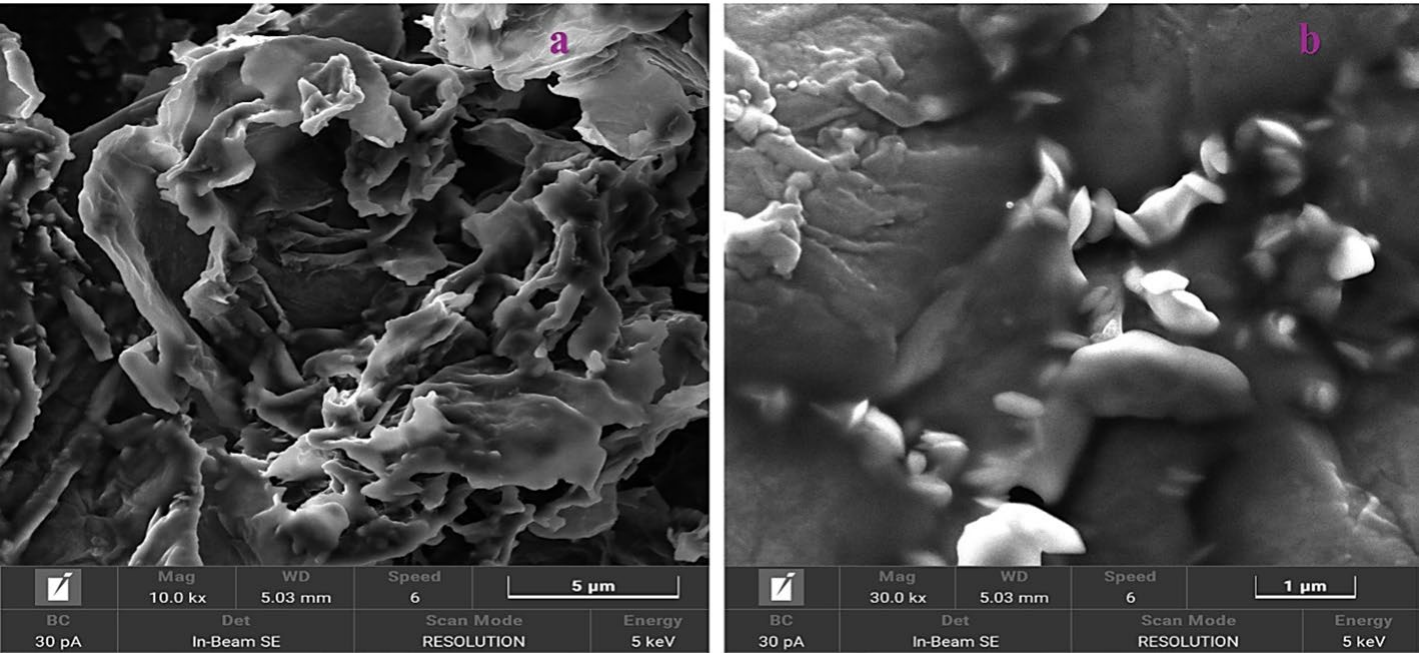
FESEM analysis of GQD synthesized nanoparticles

The crystalline structure of GQD sheets was investigated through XRD analysis, as shown in Fig. 2. It displays a prominent characteristic diffraction peak; the XRD pattern of GQD exhibits an intense (100) peak at 2θ = 43.2° with an interlamellar spacing of d = 12.11 Å. Additionally, the XRD pattern of GQD reveals a broad and less intense diffraction peak (002) at 2θ = 22.1°. The interlayer distance was determined using the Bragg equation (Eq. (2) as shown below [39]: To examine the morphology, FESEM analysis was performed using a JEOL (7610F Plus) electron microscope at GITAM Deemed to be University, Visakhapatnam. Figure 3a, b demonstrate the formation of GQDs, which appear spherical in shape at different magnifications of 5 µm and 1 µm.

2 $$ {\mathrm{2dSin}}\theta = n\lambda $$

### Engine setup

**Fig. 4:**
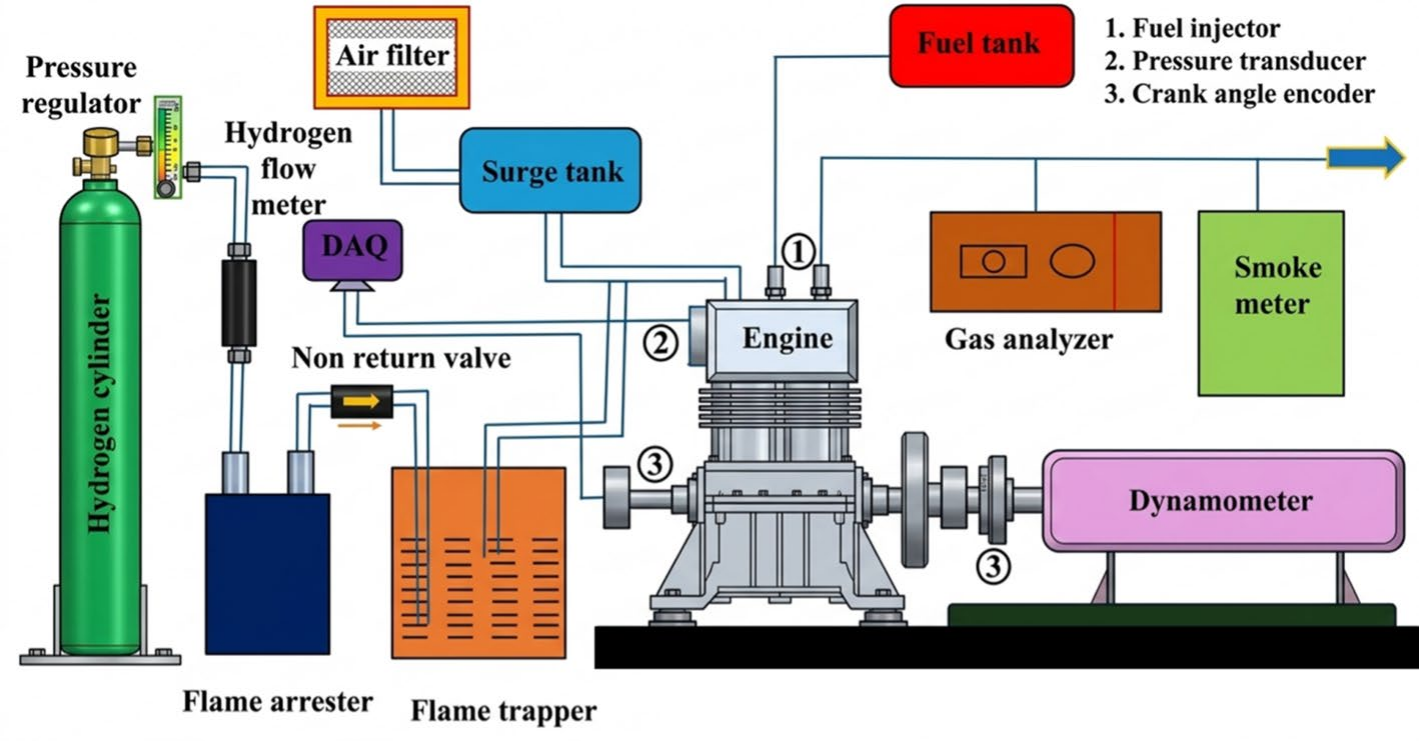
Schematic layout of the engine configuration

In the current study, a 4-stroke, 1-cylinder, DI, water-cooled, diesel engine was coupled with an eddy current dynamometer. The details of the engine configuration are mentioned in Table 4, and the schematic layout of the engine is represented in Fig. 4. Engine output parameters are measured with an AC generator at variable loads ranging from 0–100%. The engine power output is 5.7 kW at a rated speed of 1500 rpm. The fuel consumption was measured for the use of 10 cc of fuel by tracking the time. Air–fuel mixture was tracked using an orifice flow meter. The combustion parameters are obtained with the aid of crank angle encoders, which are interfaced with a data acquisition system. Exhaust gases are measured employing an AVL gas analyzer and a smoke meter. A pure hydrogen (99%) cylinder at a pressure of 160 bar is used in the study. A regulator is attached on top of the cylinder to control the quantity and supply of the gas to the inlet manifold of the engine cylinder at a 1 bar pressure through the safety devices known as flame arrestor and flame trap to prevent backfire. A timed port injection strategy was followed to mix H_2_ and intake air and supply them to the inlet and primary fuel to act as an igniter for the dual fuel operation. Initially, the diesel is fuelled and steadily operated for 30 min to reach a steady state. Then, the loads are carried, and the readings are recorded. Afterward, the prepared fuels are supplied under different loads and recorded. Finally, H_2_ is supplied along with pilot fuel, and readings are recorded under different loads. Each fuel blend is tested under different loads for 5 times, and average values are used to minimize the errors.

**Table 4:** Shows the details of the test engine

Parameter	Specification
Engine make TAF-1	Kirloskar
Rated power	5.2 kW
No. of cylinders	1
Bore/stroke in mm	100/105
compression ratio	18:1
Load	100%
Injection pressure	220 bar
Rated speed	1500 rpm

The CP was evaluated for each fuel sample under controlled conditions for 50 cycles. The mean CP was applied to find the HRR. The stability of the engine was determined by analyzing the coefficient of variation (COV_imep_), which is less than 4% under test conditions [40]. The HRR was evaluated using the following Eq. 3 [40].

3 $$ \begin{aligned} & \frac{{dQ_{n} }}{d\theta } = \frac{\gamma }{\gamma - 1} p\frac{dv}{{d\theta }} + \frac{1}{\gamma - 1}v\frac{dP}{{d\theta }} \\ & \quad = \frac{{\left( {\frac{\gamma }{\gamma - 1} \times P_{I} \times \left( {V_{i + 1} - V_{i} } \right) + \frac{1}{\gamma - 1} \times V_{I} \times \left( {P_{i + 1} - P_{i} } \right)} \right)}}{\Delta \theta } \\ \end{aligned} $$

where Q = Heat release; θ = Crank angle; V = Cylinder volume; P = In-cylinder pressure; and γ = Specific heat ratio = 1.35.

BSFC and BTE were evaluated as per Eqs. 4 and 5 shown below.

4 $$ \eta B_{th} = \frac{BP}{{m_{f} \times CV}}, $$

5 $$\mathrm{B}\mathrm{S}\mathrm{F}\mathrm{C} = \frac{SFC\times {10}^{6}}{BP}$$

where mf = mass of fuel (kg/hr); BP = Brake Power (kW); and SFC = Specific fuel consumption (kg/kW hr).

The gas analyzer generates emissions data in ppm units, which are converted to brake-specific emissions that are presented in terms of g/kWh using the following Eqs. (6,7, and 8) [1]:

For CO emissions:where ACO = 1.25 is the conversion factor.

$$ {\mathrm{CO}}\left( {{\mathrm{mg/m}}^{{3}} } \right) = {\text{CO }}\left( {{\mathrm{ppm}}} \right) \times {\mathrm{ACO}} $$

6 $$ {\mathrm{BSCO}}\,\left( {{\mathrm{g}}/{\mathrm{kWh}}} \right) = [{\mathrm{CO}}\;{\mathrm{mg/m}}^{{3}} \times {\mathrm{Air}}\;{\mathrm{volume}}\;{\mathrm{flow}}\;{\mathrm{rate}}\;{\mathrm{m}}^{{3}} {\mathrm{/h}}]{\mathrm{/BP}}\;({\mathrm{kW}}) $$

For HC emissions:where AHC = 1.965 is the conversion factor.

$$ {\mathrm{HC}}\;\left( {{\mathrm{mg/m}}^{{3}} } \right) = {\mathrm{HC}}\;\left( {{\mathrm{ppm}}} \right) \times {\mathrm{AHC}} $$

7 $$ {\mathrm{BSHC}}\;\left( {\mathrm{g/kWh}} \right) = \left[ {{\mathrm{HC}}\;\left( {{\mathrm{mg/m}}^{{3}} } \right) \times {\mathrm{Air}}\;{\mathrm{volume}}\;{\mathrm{flow}}\;{\mathrm{rate}}\;\left( {{\mathrm{m}}^{{3}} {\mathrm{/h}}} \right)} \right]{\mathrm{/BP}}\;\left( {{\mathrm{kW}}} \right) $$

For NOx emissions:where ANOx = 3.39 is the conversion factor.

$$ {\mathrm{NOx}}\;\left( {{\mathrm{mg/m}}^{{3}} } \right) = {\mathrm{NOx}}\;\left( {{\mathrm{ppm}}} \right) \times {\mathrm{ANOx}} $$

8 $$ {\mathrm{BSNOx}}\;\left( {{\mathrm{g}}/{\mathrm{kWh}}} \right) = \left[ {{\mathrm{NOx}}\;\left( {{\mathrm{mg/m}}^{{3}} } \right) \times {\mathrm{Air}}\;{\mathrm{volume}}\;{\mathrm{flow}}\;{\mathrm{rate}}\;\left( {{\mathrm{m}}^{{3}} {\mathrm{/h}}} \right)} \right]{\mathrm{/BP}}\;\left( {{\mathrm{kW}}} \right) $$

### Uncertainty of the measurements

The experimental investigation results must be produced accurately. Otherwise, these may affect different types of errors in which the uncertainty analysis is required to perform to evaluate the accuracy of the results. Standard protocols exist to quantify the errors during experimentation. These errors may be obtained from different sources. For instance, human errors in reading, ecological errors, and instrumental errors. And standardization error. The uncertainty of the measurements is found by employing the root sum squares method. The overall uncertainty is determined using the following Eq. 9 [41]. The % of uncertainty measurements for various parameters is shown in Table 5.

9 $$ {\mathrm{U}}_{{\mathrm{E}}} = \sqrt {\mathop \sum \limits_{j = 1}^{n} X_{j}^{2} } $$

**Table 5:** Precision and the uncertainty of measuring parameters

Sl. No	Instrument /devise	Range of measurement	Precision	% of uncertainties
1	BTE	–	–	± 0.75
2	BSFC	–	–	± 0.5
3	CO	0–10 Vol.%	± 0.01 Vol. %	± 1
4	HC	0–30,000 ppm	± 15 ppm Vol	± 1
5	NOx	0–4800 ppm	± 10 ppm	± 1.2
6	Smoke meter	–	± 1	± 1.0
7	Crank angle	0–720 ℃A	± 0.2℃A	± 0.4
8	HRR	–	–	± 0.6
9	Speed	0–10,000 rpm	± 5 rpm	± 0.2

For this current investigation, the uncertainties of BTE, BSFC, CO, HC, NOx, and Smoke are termed as X_1_, X_2_, X_3_….X_6_, respectively, as shown in Eq. 10.

10 $$ \begin{gathered} {\mathrm{U}}_{{\mathrm{E}}} = \sqrt {X_{1}^{2} + X_{2}^{2} + X_{3}^{2} + X_{4}^{2} + X_{5}^{2} + X_{6}^{2} } \hfill \\ {\mathrm{U}}_{{\mathrm{E}}} = \sqrt {{0}{\mathrm{.75}}^{{2}} { + 0}{\mathrm{.5}}^{{2}} { + 1}^{{2}} { + 1}^{{2}} { + 1}{\mathrm{.2}}^{{2}} { + 1}^{{2}} } \hfill \\ {\mathrm{U}}_{{\mathrm{E}}} = { 2}.{29}\% \hfill \\ \end{gathered} $$

Thus, the overall uncertainty of the experiments is found to be ± 2.29%. This analysis is meant for the errors of environmental conditions, human errors, calibration errors, and instrumental errors, ensuring the results are accounted for at the appropriate level of accuracy.

## Results and discussions

### Performance parameters

#### Brake thermal efficiency

**Fig. 5:**
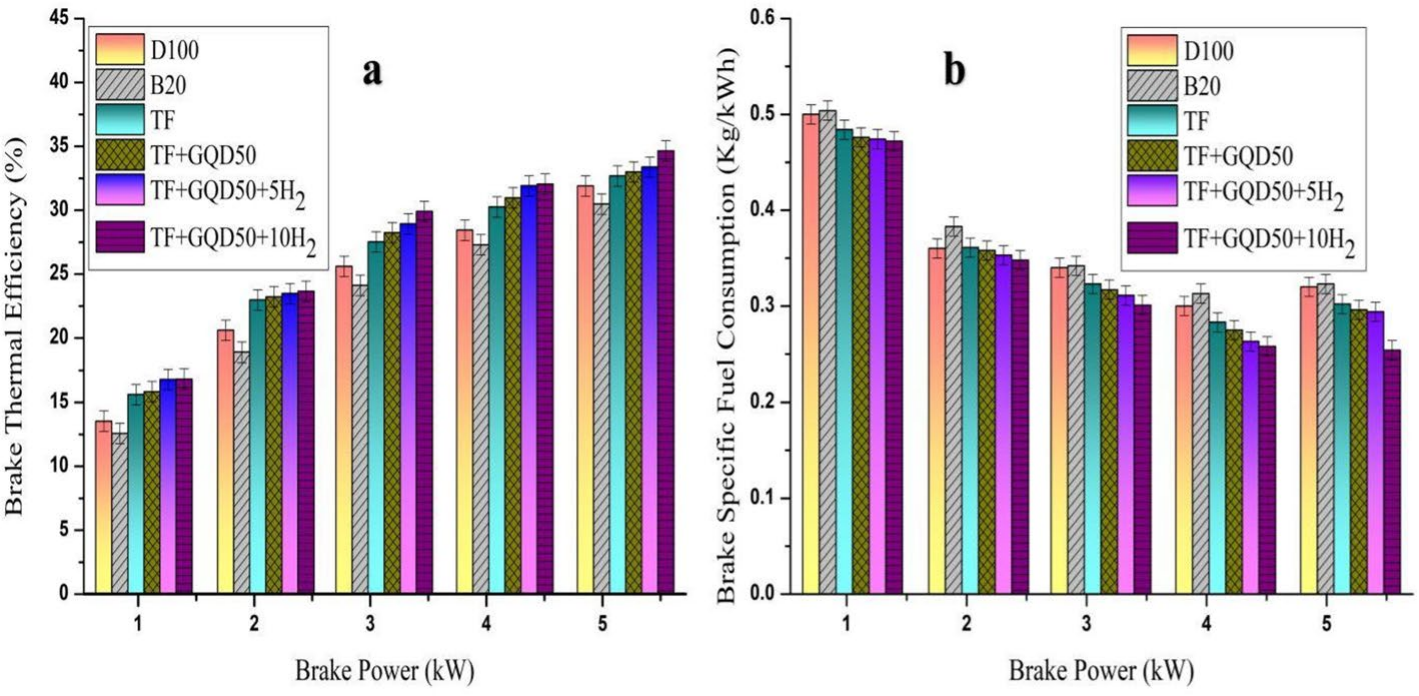
Presents **a** BTE versus BP, and **b** BSFC versus BP for various tested fuels

Figure 5a depicts the effect of BTE vs BP for the used blends. BTE is defined as the fuel energy transferred into actual power. The BTE values are found to be 31.8, 30.6, 32.4, 33.02, 33.5, and 34.8% for D100, B20, TF, TF + GQD50, TF + GQD50 + 5H_2_, and TF + GQD50 + 10H_2_ individually at maximum BP. The maximum increment in BTE is observed to be 13.7% for the TF + GQD50 + 10H_2_ sample compared to the B20 mix at higher load. BTE is observed to be low for B20 due to low heating value and greater viscosity, which tend to cause improper mixing and poor atomization. Thereby, combustion is slow and has low performance. The TF sample results in better performance than D100, owing to better spray properties, vaporization characteristics, and oxygen content. Moreover, the NPs included TF sample enhanced further due to higher thermal conductivity of NPs, thermal diffusion, improved rate of reaction in the pre-combustion zone, and shortened ID. Nevertheless, the infusion of H_2_ results in maximum BTE values due to superior heating value, faster flame speed, and rapid combustion. In another investigation, Xia et al. [42] noticed that the alumina-doped (at a dosage of 25 and 50 mg/l) caster biodiesel blends, along with H_2_ infusion at a fraction of 25%, showed improved BTE by 2.5%, which is due to higher energy in H_2_, a higher rate of reaction due to the inclusion of NPs, and efficient combustion. In another article, Dhamodaran et al. [43] studied the addition of a Manilkara zapota oil-based B30 blend with Al_2_O_3_ emulsified NPs at dosages of 50 and 100 mg/l, and H2 at a flow rate of 10 lpm, and tested it on a dual-fuel diesel engine. The findings disclosed that the BTE is raised by 29.66%, which is ascribed to the combined effects of Al_2_O_3_ NPs and hydrogen addition, which enhance combustion by reducing ID, increasing peak CP and HRR, and promoting faster, more complete burning. Further, Nanoparticles act as catalysts and improve atomization and mixing. Hydrogen provides high flame speed for better premixed combustion and energy release.

#### Brake specific fuel consumption

Figure 5b depicts the BSFC vs BP for the fuel mixtures. The BSFC is said to be the amount of fuel disbursed to produce a unit of BP. The BSFC values are observed to be 0.336, 0.342, 0.329, 0.326, 0.324, and 0.272 kg/kWh for D100, B20, TF, TF + GQD50, TF + GQD50 + 5H_2_, TF + GQD50 + 10H_2_, respectively, at higher BP. The BSFC is reduced by 20.46% for the TF + GQD50 + 10H_2_ sample at maximum BP. The large surface area and thermal properties of NPs increase the homogeneity of combustion, assuring maximum energy release during the expansion process. Thereby, reducing fuel consumption by a self-governing technique which states that when a sufficient amount of energy is released during combustion, the CP reaches its maximum peak point and starts expanding immediately, thereby cutting off the fuel. The synergistic effect of NPs, higher oxygen levels in the alcohol, and higher energy content of H_2_ facilitates faster evaporation, rapid combustion, and lower BSFC. In an article, Zhang et al. [36] concluded that the addition of TiO_2_ NPs at a concentration of 100 mg/l in WCO-diesel blends along with H_2_ infusion resulted in lower BSFC values by 5–15%, which is owing to the higher calorific value of H_2_, the reaction rate, which promotes accelerated combustion and reduced BSFC. In another study, Premkumar et al. [44] reported that the inclusion of hybrid NPs (CeO_2_ + Al_2_O_3_ and GO + CNTs) at a dosage of 100 mg/l in microbial oil biodiesel with H_2_ infusion at a supply rate of 5 lpm was tested on a dual-fuel engine. The BSFC is reduced by 12.21% for metal-based NPs and 15.02% for carbon-based NPs, as correlated to diesel at higher load. The lower BSFC of metal-based NPs and H2-enriched blends leads to higher flame speed of H_2_, better air–fuel interaction, thermal diffusivity of NPs, which shortens the ID, and combustion. While the carbon-based hybrid NPS with H_2_ addition leads to superior thermal conductivity of NPs, the thermal recovery mechanism, enhanced oxidation, and higher energy content of H_2_ lead to enhanced combustion and performance characteristics.

### Combustion characteristics

#### Cylinder pressure (CP)

**Fig. 6:**
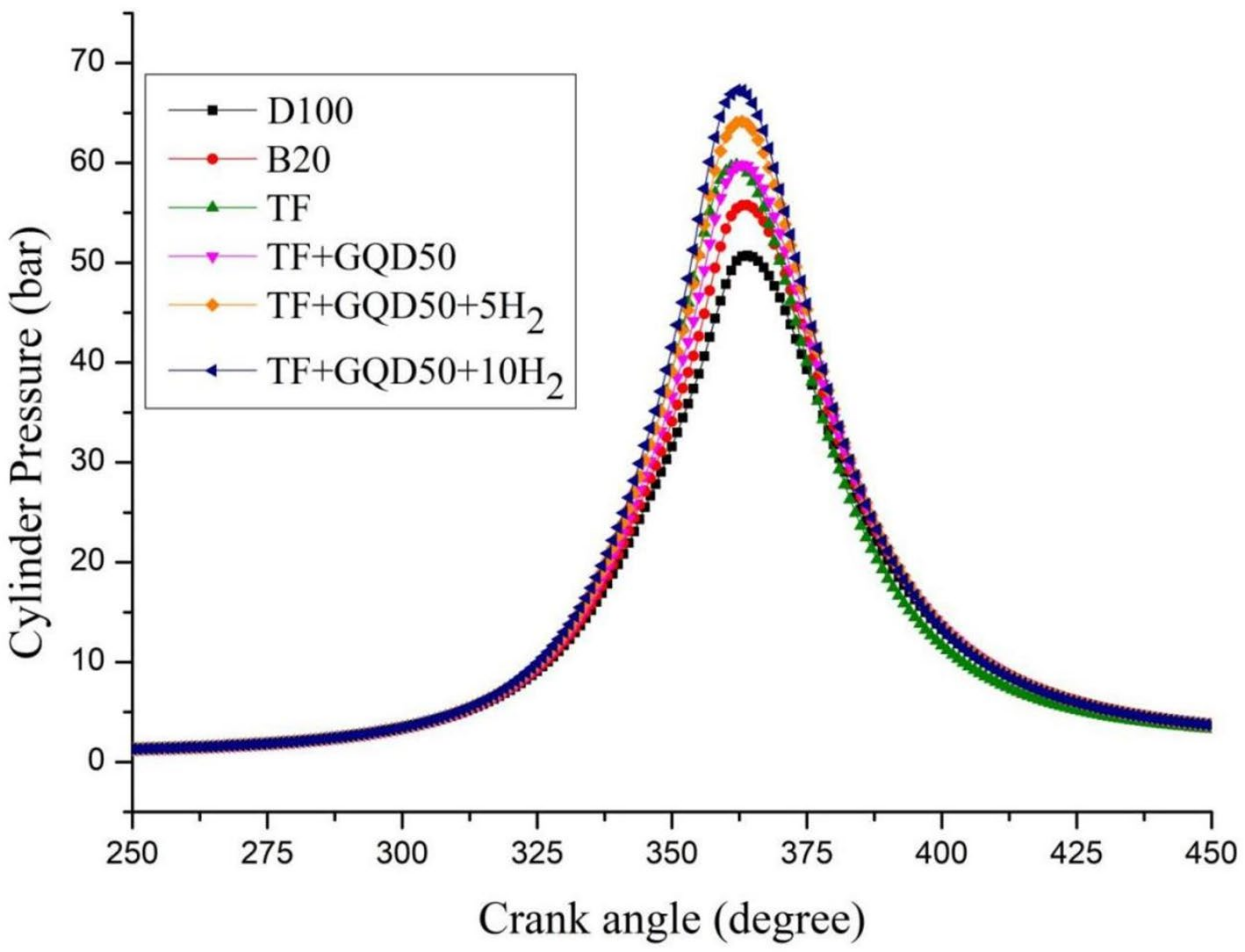
Illustrates the cylinder pressure versus crank angle for different fuel mixtures

The energy release and combustion efficiency are presented by the cylinder pressure. Figure 6 depicts the variation of CP vs. crank angle for the tested fuel samples. The CP values are found to be 51.4, 56.7, 59.4, 59.6, 64.4, and 66.6 bars for D100, B20, TF, TF + GQD50, TF + GQD50 + 5H_2_, and TF + GQD50 + 10H_2_, respectively, at maximum BP. The CP value is poor for D100 and B20, which are established as base references. The CP values are observed to be 59.4 and 59.6 bars, which are close to each other. These values are noted to be higher than those of diesel and B20 samples due to higher oxygen levels in alcohol and NPs additives, improved volatility, and rate of reaction, which aid in accelerating combustion. Moreover, the CP values reach their highest peak point (64.4 and 66.6 bars) for TF + GQD50 + 5H2 and TF + GQD50 + 10H2 samples, which is improved by 17.46 and 29.4% compared to the diesel fuel at higher BP. These results are mainly attributed to the superior thermal conductivity of NPs, micro-explosion, and improved surface area, which directly affect the fuel evaporation and atomization, thus facilitating the achievement of maximum combustion and CP [45]. Considering the above phenomenon, when H_2_ is used in conjunction with pilot fuel, the combustion is further accelerated due to its superior energy density [44]. In a recent study, Das and Das [46] showed that the addition of iron NPs at a quantity of 75 mg/l in WCO mixtures, along with H_2_ infusion at 10 lpm, resulted in improved CP by 5.3%, which is due to enhanced air–fuel interactions, evaporation, enhanced rate of reaction, and 3 floods of higher energy of H_2_. In another study, Gurusamy and Ponnusamy [47] explored the addition of DEE at volumes of 10 and 20% in camphor oil-based biodiesel blends (B30) in conjunction with H_2_ at a supply rate of 8 lpm on a dual-fuel engine. The combustion parameters reported that the CP has reached its peak value with NPs inclusion in pilot fuel and H_2_ enrichment. The CP value is enhanced by 6.83%, which is attributed to higher heating value, maximum flame velocity, and greater thermal properties of NPs.

#### Heat release rate (HRR)

**Fig. 7:**
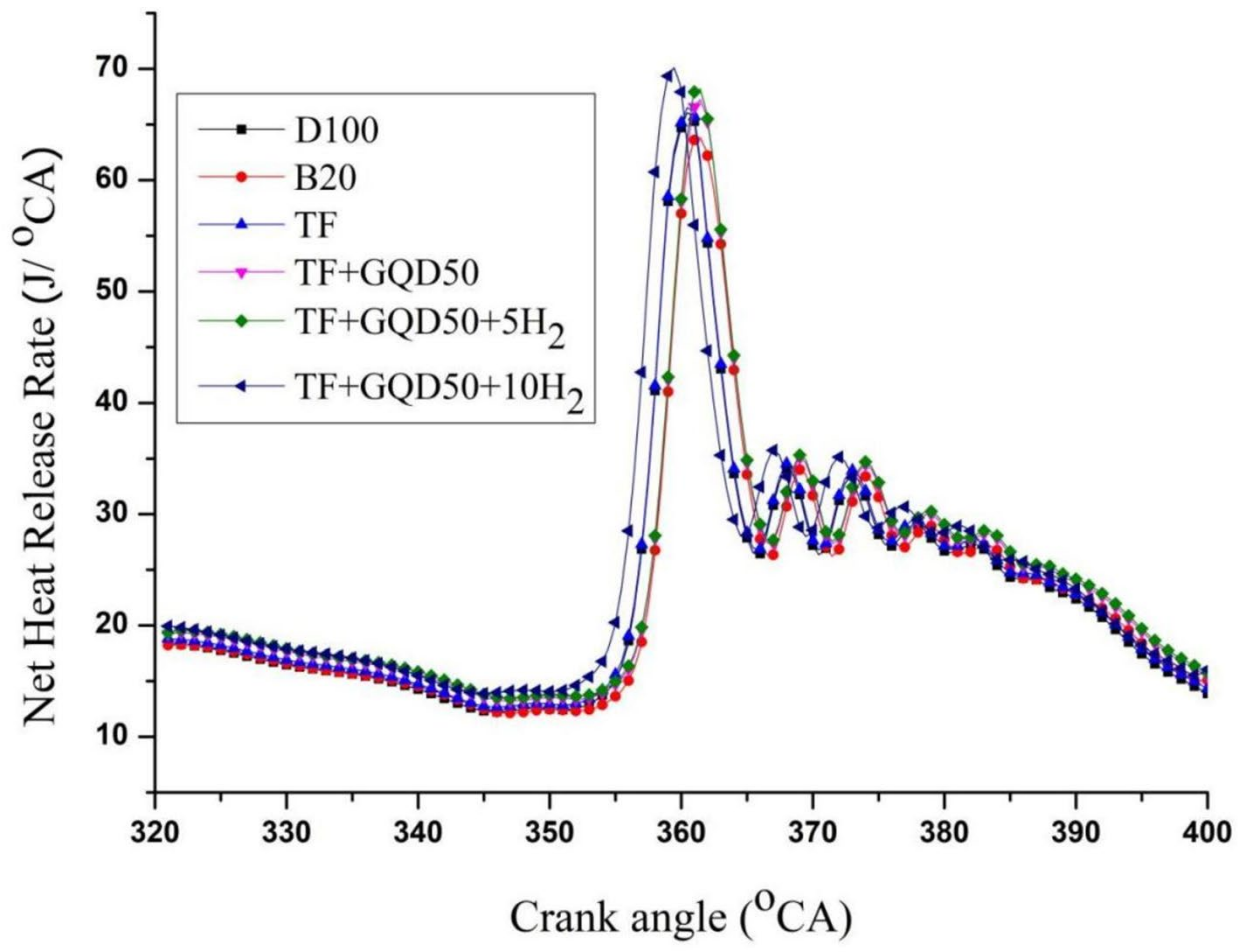
Depicts the effect of various fuel samples on HRR with reference to crank angle

The HRR indicates insights into energy conversion and combustion dynamics. The variation of HRR vs Crank angle for the tested fuel samples is shown in Fig. 7. It is observed from the figure that the HRR values are found to be 65.16 and 64.32 J/℃A for D100 and B20 samples. The B20 sample’s poor calorific value and higher viscosity are responsible for the slow combustion process. Thereby, HRR is found to be lower than the D100. Further, HRR values are raised to 65.25 and 65.82 J/℃A for TF and TF + GQD50 samples. The oxygen level, higher cetane number, higher thermal conductivity, and improved reaction rate are the reasons for the HRR improvement. Moreover, the HRR is found to be 66.34 and 67.21 J/℃A for H_2_ infused along with the TF + GQD50 sample at a flow rate of 5 and 10 lpm, respectively, at higher load conditions. This enhancement is mainly owing to the superior energy level of H_2_ and the faster flame velocity. The maximum HRR is increased by 4.49% for the TF + GQD50 + 10H_2_ sample, which is due to the higher flow rate of H_2_. In an investigation, Pullagura et al. [48] demonstrated that the dispersion of GNPs at a dosage of 50 ppm, and n-Butanol at a vol. of 10% in Sterculia foetida oil-based B20 as a pilot fuel, and H_2_ at a rate of 6 lpm as a supplementary fuel with air at the inlet manifold resulted in improved HRR by 6.23% as compared to the B20 mix at maximum load. The improvement in HRR is due to greater thermal properties of NPs, greater flammability, superior calorific value of H_2_, and shorter ID.

### Emission characteristics

#### Carbon monoxide (CO)

**Fig. 8:**
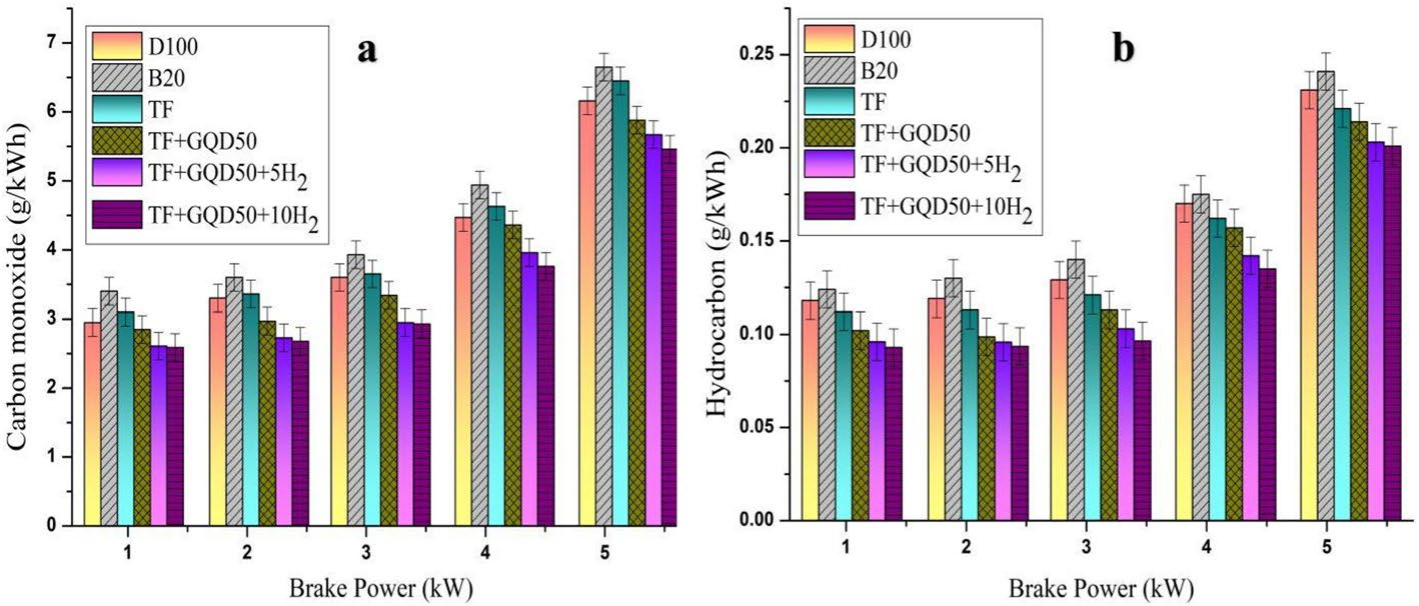
Illustrates **a** carbon monoxide versus BP, and **b** hydrocarbon versus BP for the prepared mixtures

Figure 8a shows the effect of CO gas vs BP for the prepared fuel mixtures. The CO emissions are emitted through incomplete combustion, poor evaporation, and atomization. The addition of DEE improves cetane number, which enhances the volatility, atomization, spray properties, and oxygen stability, leading to efficient combustion. While the GNP NPs enhance the thermal properties and rate of reaction, which facilitates proper combustion and reduces CO emissions. Nevertheless, the H_2_ enrichment as a secondary fuel, which possesses a higher heating value, faster flame speed, and quicker combustion. Thus, leading to complete combustion and a decrease in CO emissions. The CO values are noted to be 6.22, 6.62, 6.45, 5.84, 5.68, 5.46 g/kWh for D100, B20, TF, TF + GQD50, TF + GQD50 + 5H_2_, TF + GQD50 + 10H_2_, respectively, over the B20 sample at higher BP. The maximum reduction in CO emission is found to be 17.52% compared to the B20 mix. In a similar article, Jayabal et al. [49] disclosed that the addition of GO NPs at a concentration of 50 mg/l and bio-ethanol at a vol. of 10% in algae biodiesel and H_2_ enrichment at a supply rate of 3 and 6 lpm as a supplementary fuel resulted in decreased CO emission by 57.75% and 77.58%, respectively, at maximum load, which is ascribed to greater flame speed, oxygenated alcohol and NPs, and rapid combustion.

#### Hydrocarbon emission (HC)

The HC emissions are produced similarly to the influence parameters of CO. The unburnt HC is emitted due to poor air–fuel intermingling, accumulation of fuel particles due to higher viscosity and poor evaporation, and incomplete combustion. Figure 8b shows the effect of HC emissions vs BP for various prepared mixtures. The HC gases are noticed to be 0.227, 0.239, 0.222, 0.218, 0.209, and 0.202 for D100, B20, TF, TF + GQD50, TF + GQD50 + 5H_2_, TF + GQD50 + 10H_2_, respectively, over the B20 sample at maximum BP. The highest fall in HC emission is 11.01% for TF + GQD50 + 10H_2_ which is ascribed to greater rate of H_2_ supply and its energy content, improvement of oxygen content in the overall blend, large surface area, and catalytic effect [32]. An article explored by Bikkavolu et al. [50] revealed that the inclusion of Boron nitride NPs at a quantity of 50 mg/l and DMC at a vol. of 10% in neem oil-based B20 sample, in conjunction with H_2_ supply at 8 lpm, showed a mitigation of HC emission by 16.6%, which is due to the synergistic effect of H_2_, the oxidation capability of NPs, and higher atomization and oxygen content of DMC alcohol.

#### Nitrogen oxides (NOx)

**Fig. 9:**
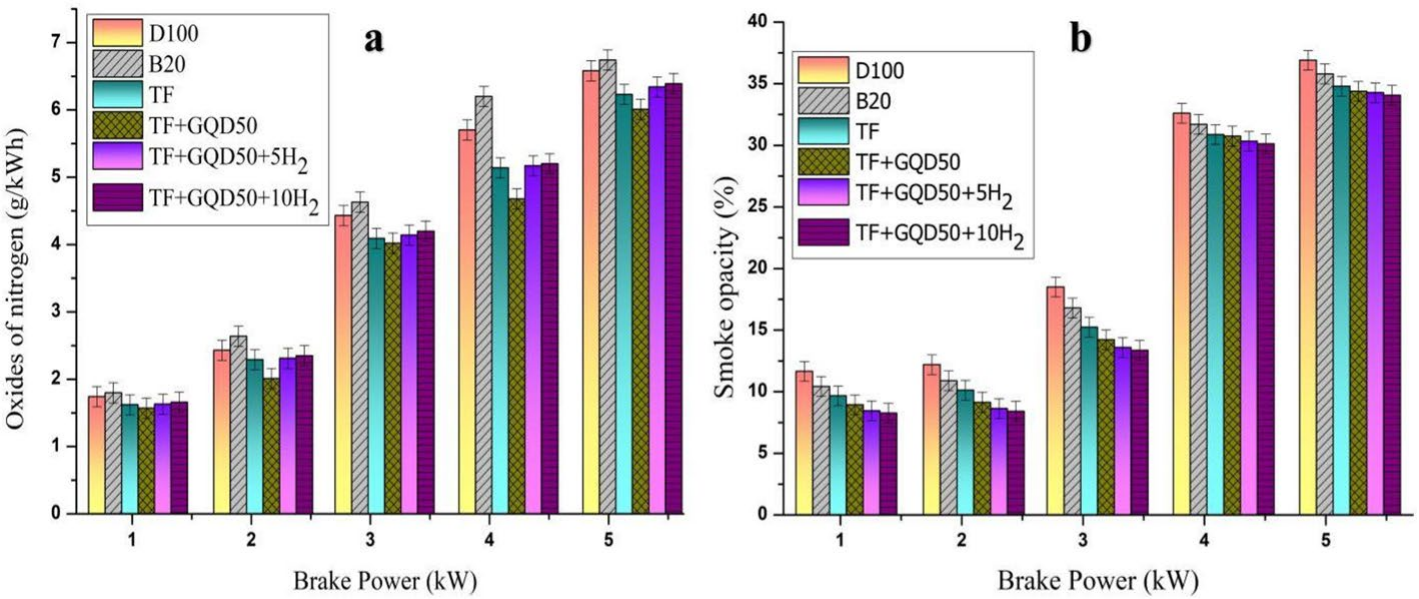
**a** Oxides of nitrogen versus BP, and **b** smoke opacity versus BP for different fuel mixtures

The NOx emission is mainly emitted due to combustion duration/residence time and cylinder temperature. Figure 9a illustrates the effect of NOx vs BP for the experimental samples. NOx is found to be 6.51 and 6.62 g/kWh for D100 and B20 mix at maximum BP, which are found to be higher compared to other samples. The higher NOx is due to the greater oxygen level of biodiesel, higher turbulence in the combustion zone at higher BP. Moreover, the NOx results are observed to be decreased to 6.24 and 6.16 g/kWh for TF and TF + GQD50 samples at higher BP, owing to the quenching effect of alcohol and the heat absorption capacity//latent heat of evaporation of the NPs. The maximum reduction in NOx emissions is found to be decreased by 5.74, and 6.94% for TF and TF + GQD50 samples, as compared to the B20 sample, which is due to the combined effects of improved combustion efficiency, charge dilution, and optimized operating conditions, which may also contribute to NOx reduction [51]. While NOx emission is slightly increased to 6.30 and 6.36 g/kWh for H2 infused blends at a rate of 5 and 10 lpm, as compared to TF and TF + GQD50 samples, respectively, which is ascribed to higher calorific value, greater flame temperature, and rapid combustion. The enrichment of H_2_ in the TF + GQD50 mix at a rate of 10 lpm reduced NOx by 3.9% as compared to the B20 mix. In a study, Pullagura et al. [52] stated that the inclusion of GO NPs at a concentration of 50 mg/l in Nerium oleander oil blends (B20) as a pilot fuel and an H_2_ supply of 10 lpm revealed improved NOx emissions by 11.98% due to the superior calorific value of H_2_ and the faster burning rate of the fuel mix with H_2_. In another article, Mohanraj et al. [53] showed that the addition of GO NPs at a dosage of 50 mg/l, and bio-ethanol at a vol. of 10% in algae biodiesel along with H_2_ at a supply rate of 3 and 6 lpm resulted in improved NOx emission by 20.7% and 39% which is due to greater combustion reaction, and faster burning of H_2_.

#### Smoke opacity

Figure 9b shows the smoke opacity of various blends with respect to the BP. The smoke opacity values are increased as the BP rises. The smoke opacity results are found to be 33.2, 33.1, 32.9, 32.78, 32.65, and 32.46% for D100, B20, TF, TF + GQD50, TF + GQD50 + 5H_2_, TF + GQD50 + 10H_2_, respectively, at maximum BP. The greatest reduction of smoke opacity is observed to be 2.22% compared to that of diesel. The oxygen level in the fuel mixtures, better air–fuel mixing, evaporation, improved reaction rate with NPs, higher flammability of H_2_, which favors the combustion process, and reduced smoke opacity [50]. In a similar article, Liu et al. [54] reported that the addition of butanol at a vol. of 10%, Copper oxide (CuO) NPs at a dosage of 100 mg/l in a Murraya Koenigii-based B20 sample, and H_2_ at a flow rate of 10 lpm supplied as a secondary fuel resulted in lowered smoke opacity due to higher thermal diffusivity, faster flame propagation, oxygenated alcohol addition, catalytic effect of NPs, and improved combustion efficiency. The performance, combustion, and emission characteristics of various studies are compared with the present investigation and are given in the Table 6.

**Table 6:** Shows the performance, combustion, and emission characteristics of previous investigations compared with the current study

Sl. no	Engine details	Base fuel	NPs/alcohols used	Flow rate of H_2_	Performance parameters	Combustion parameters	Emission parameters	References
1	Single-cylinder, 4-stroke, at a rated speed of 1500 rpm, Kirloskar, model TV1, 5.2 kW, attached with Eddy current dynamometer, Dual fuel, CI engine	Madhuca oil-based Biodiesel-Diesel blend (B20)	TiO_2_—50, and 100 ppm	10 lpm	BTE ↑24.23%, BSFC ↓8.32%	CP ↑17.7%, HRR ↑4.65%	HC ↓13.16%, CO ↓19.02%, Smoke ↓4.04%, and NOx ↑ 12.67%	[20]
2	Single-cylinder, 4-stroke, at a rated speed of 1500 rpm, Kirloskar, model TV1, 5.2 kW, Dual fuel, CI engine	Jatropha oil-based biodiesel mixtures (B20)	Al_2_O_3_, TiO_2_, and MWCNTs—60 mg/l, and Heptane—10% Vol	6 lpm	BTE ↑10.9%, BSFC ↓16.6%	CP ↑ 33.3%, HRR ↑9.14%	HC ↑10.8%, CO2 ↓10.7%, CO ↑15.4%, Smoke ↓31%, and NOx ↑	[30]
3	Single-cylinder, 4-stroke, at a rated speed of 1500 rpm, Kirloskar, model TV1, 5.2 kW, Dual fuel, CI engine	Manilkara zapota methyl ester (M30)	Al_2_O_3_—50, and 100 ppm	10 lpm	BTE ↑29.66%, BSFC ↓6.2%	CP ↑27.31%, HRR ↑5.3%	HC ↓10.6–12%, CO ↓20%, Smoke ↓15%, and NOx ↑6.07%	[43]
4	Single-cylinder, 4-stroke, at a rated speed of 1500 rpm, Kirloskar, model TV1, 5.2 kW, Dual fuel, CI engine	Sterculia Foatida oil based Biodiesel-Diesel blend (B20)	GO—50 mg/l, and n-Butanol—10% Vol	3 and 6 lpm	BTE ↑7.54%, BSFC ↓20.75%	CP ↑9.8%, HRR ↑6.23%	HC ↓24.86%, CO ↓19.75%, Smoke ↓8.8%, and NOx ↓4.4%	[48]
5	Single-cylinder, 4-stroke, at a rated speed of 1500 rpm, Kirloskar, model TV1, 5.2 kW, Dual fuel, CI engine	Neem oil based Biodiesel-Diesel blend (B20)	Boron Nitride—50 mg/l, and Dimethyl Carbonate—10% Vol	8 lpm	BTE ↑16.4%, BSFC ↓22.2%	CP ↑17.7%, HRR ↑4.65%	HC ↓16.6%, CO ↓14.8%, Smoke ↓12.9%, and NOx ↑ slightly	[50]
6	4-stroke, single-cylinder, Kirloskar TV1 at a rated power output of 5.2 kW, Duel fuel, CI Engine	Colza biodiesel, Margosa biodiesel, and blends of both biodiesels	–	10 lpm	BTE ↑27.02%, BSFC ↓10%	–	HC ↓10–12%, CO ↓8–10%, Smoke ↓15%, and NOx ↑slightly	[55]
7	Single-cylinder, 4-stroke, at a rated speed of 1500 rpm, SV1 & Kirloskar, 5.9 kW, Dual fuel, CI engine	Jamun oil based B20	Ethanol—5, and 10% Vol	4 lpm	BTE ↑7.55%, BSFC ↑ marginally	CP ↑ 14.2%, HRR ↑7.40%	HC ↑25%, CO ↑23.1%, Smoke ↓31%, and NOx ↑8.2%	[56]
8	Single-cylinder, 4-stroke, at a rated speed of 1500 rpm, Kirloskar, model TV1, 4.4 kW, Dual fuel, CI engine	Palmyra oil based biodiesel-Diesel blends (B20)	TiO_2_—50, and 100 ppm	0.727 lpm	BTE ↑5.5%, BSFC ↓5.5%	CP ↑5.95%, HRR ↑7.6%	HC ↓21.9–12%, CO ↓11.8%, Smoke ↓15.5%, and NOx ↑15.8%	[57]
9	Single-cylinder, 4-stroke, at a rated speed of 1500 rpm, Kirloskar, model TV1, 5.2 kW, Dual fuel, CI engine	Nerium Oleander oil based Biodiesel-Diesel blend (B20)	MWCNTs—50 mg/l, and ethanol—10% Vol	6 lpm	BTE ↑7.79%, BSFC ↓17.6%	CP ↑ 13.5%, HRR ↑5.9%	CO_2_ ↑8.5%, CO ↓10.2%, and NOx ↓8.2%	[32]
10	Single-cylinder, 4-stroke, at a rated speed of 1500 rpm, Kirloskar, model TV1, 5.2 kW, Dual fuel, CI engine	WCO based B20	GQD—50 mg/l, and Ditheyl Ether—10% Vol	5, and 10 lpm	BTE ↑13.7%, BSFC ↓20.46%	CP ↑29.47%, HRR ↑4.49%	CO ↓17.52%, HC ↓11.01%, NOx ↓3.9%, Smoke ↓2.22%	Current study

## Conclusions

The present investigation focused on the influence of DEE alcohol at a vol. of 10%, and carbon-based GQD NPs dispersion in WCO-based B20 sample, and H2 enrichment at a flow rate of 5 and 10 lpm to evaluate the performance, combustion, and emission parameters of a dual-fuel CI engine. The D100, TF, and TF + GQD50 samples are supplied as a pilot fuel. While H_2_ is infused along with air as a supplementary fuel, TF + GQD50 is fuelled to the injector as a pilot fuel sample. The following inferences are drawn based on the discussions:

- The TF and TF + GQD50 samples are observed to be homogeneous and consistent. The homogeneity of the fuel mixture TF + GQD50 is confirmed by characterization.
- The performance characteristics are noticed to be improved, such as BTE is enhanced by 13.7%, and BSFC is reduced by 20.46% for TF + GQD50 + 10H_2_ as correlated to the B20 sample at higher BP, which is attributed to enhanced evaporation, higher thermal characteristics, catalytic activity, and higher energy density of H_2_.
- Moreover, the combustion parameters, including CP and HRR, reach their peak values and are improved by 29.47 and 4.49% for the TF + GQD50 + 10H_2_ sample at higher BP. This enhancement is attributed to the catalytic effect of NPs, the higher cetane number of alcohols, and the faster flame speed of H_2_.
- Further, the emissions, such as CO, are decreased by 17.5% for TF + GQD50 + 10H_2_ when correlated to the B20 sample. While HC and smoke opacity are mitigated by 11.01 and 2.22% for the TF + GQD50 + 10H_2_ sample over D100. The lower CO, HC, and smoke opacity emissions are due to superior oxygen content, better heat diffusion, catalytic activity of NPs, and higher heating value of H_2_.
- On the other side, NOx emission is shown to have a better decrement by 5.74 and 6.94% for TF and TF + GQD50 mix at higher BP. Nevertheless, the NOx emission is slightly raised for H2-infused blends, which are less than the D100 and B20 samples. While the heat absorption capability of NPs and the quenching process of alcohol favored reducing cylinder temperature, resulting in lower NOx emission.

The above inferences showed that the effect of 10% vol. of DEE, 50 mg/l GQD NPs in B20, and H_2_ infusion at a rate of 5 and 10 lpm, along with the air at the inlet manifold, enhanced the performance, combustion, and emission parameters. Thereby, the fuel sample TF + GQD50 + 10H_2_ was demonstrated to be a promising fuel and suggested to be used in short-term performance and emissions benefits in a dual-fuel CI engine operation with minimal modifications. However, long-term durability, component wear, deposit formation, lubrication effects, hydrogen safety, and engine life require further investigation before practical implementation.

## Limitations and practical considerations

The study is limited to short-term engine testing and does not evaluate long-term durability, fuel stability, nanoparticle emissions, deposit formation, lubricant degradation, or hydrogen safety. Practical implementation requires further techno-economic and lifecycle assessments.

### Future scope

From the above investigation, the fuel mixture TF + GQD50 + 10H_2_ is suggested to be used in a dual-fuel diesel engine. The recommended fuel sample can be used in the same engine and tested under different input parameters, such as Injection Timing, Injection Pressure, and Compression Ratio, to optimize the performance, combustion, and emission characteristics.

## Data Availability

The datasets used and/or analysed during the current study available from the corresponding author on reasonable request.

## Abbreviations

Al_2_O_3_: Aluminium oxide
ASTM: American Society for Testing and Method
B20: 20% Volume of biodiesel is mixed with 80% volume of diesel
BP: Brake power
BSFC: Brake specific fuel consumption
BTE: Brake thermal efficiency
CeO_2_: Cerium oxide
CI: Compression ignition
CNG: Compressed natural gas
CNTs: Carbon nanotubes
CO: Carbon monoxide
CP: Cylinder pressure
CuO: Copper oxide
DEE: Diethyl ether
DI: Direct injection
DMC: Dimethyl carbonate
FESEM: Field emission scanning electron microscopy
FFA: Free fatty acids
FTIR: Fourier transform infrared spectroscopy
GNPs: Graphene nanoplatelets
GO: Graphene oxide
GQD: Graphene quantum dots
H_2_: Hydrogen
HC: Hydrocarbon
HRR: Heat release rate
HRTEM: High-resolution transmission electron microscopy
ID: Ignition delay
IEA: International Energy Agency’s
KOH: Potassium hydroxide
LPG: Liquid petroleum gas
MWCNT: Multi wall carbon nanotubes
NOx: Nitrogen oxide
NPs: Nanoparticles
SI: Spark ignition
SiO_2_: Silicon dioxide
STEPS: Stated policies scenario
TF: Ternary fuel
TF + GQD50: 50 Mg/l of GQD dispersed in TF sample
TF + GQD50 + 5H_2_: 5 Lpm of H2 is supplied with pilot fuel TF + GQD50
TF + GQD50 + 10H_2_: 10 Lpm of H2 is supplied with pilot fuel TF + GQD50
TiO_2_: Titanium dioxide
WCO: Waste cooking oil
WCOME: Waste cooking oil methyl ester
ZnO: Zinc oxide
